# Identifying a growth and survival bottleneck: oceanic zooplankton abundance and Faroe shelf primary production jointly influence the survival of Faroe Plateau cod larvae

**DOI:** 10.1093/plankt/fbaf018

**Published:** 2025-05-15

**Authors:** Sólvá Jacobsen, Helga Bára Mohr Vang, Sólvá Káradóttir Eliasen, Karin Margretha Húsgarð Larsen, Eilif Gaard, Peter Grønkjær, Hjálmar Hátún

**Affiliations:** Department of Marine Environment, Faroe Marine Research Institute, Nóatún 1, 100 Tórshavn, Faroe Islands; Department of Marine Resources, Faroe Marine Research Institute, Nóatún 1, 100 Tórshavn, Faroe Islands; Department of Marine Resources, Faroe Marine Research Institute, Nóatún 1, 100 Tórshavn, Faroe Islands; Department of Marine Environment, Faroe Marine Research Institute, Nóatún 1, 100 Tórshavn, Faroe Islands; Department of Marine Environment, Faroe Marine Research Institute, Nóatún 1, 100 Tórshavn, Faroe Islands; Department of Biology, Aquatic Biology, Aarhus University, Ole Worms Allé 1, 8000 Aarhus C, Denmark; Department of Marine Environment, Faroe Marine Research Institute, Nóatún 1, 100 Tórshavn, Faroe Islands

**Keywords:** cod, growth, survival, Faroe shelf, *Calanus finmarchicus*, larvae, juveniles, primary production index

## Abstract

The survival of Faroe Plateau cod larva to the juvenile stage varies significantly between years, impacting recruitment. Zooplankton abundance is likely a key driver in this variability, but to what extent specific prey species are key to survival of the larvae has been unclear. This study explores a seasonally resolved zooplankton dataset from a high-recruitment year (2017) and two following low-recruitment years (2018 and 2019) collected along a hydrographic/biological transect extending from the central shelf to the open ocean west of the Faroes; a key region, with strong influxes of oceanic waters to the shelf. The aim was to identify zooplankton species crucial for cod larval growth and survival. Analyses suggest a positive relationship between pelagic juvenile indices and primary production on the central shelf as well as the oceanic abundance of the copepod *Calanus finmarchicus*. Using partial least square regression on previously unpublished longer term data on copepod abundances in the upper layer in the Faroe Bank Channel and a primary production index for the Faroe shelf, we found that the shelf primary production combined with the abundance of late-stage *C. finmarchicus* in oceanic waters in May account for half of the interannual variability in juvenile cod abundance.

## INTRODUCTION

Wide distribution and high abundance of pelagic 0-group fish, commonly referred to as larval and juvenile fish in their earliest life stages, play a crucial role in the transfer of energy between different trophic levels in coastal shelf ecosystems (Eriksen *et al.,* 2011). Furthermore, for species like cod (*Gadus morhua*), larval and juvenile growth and survival are considered essential for determining the final year-class strength (Leggett and Deblois, 1994). A key factor in survival of fish larvae is rapid growth through the early stages of development when vulnerability to predation, starvation and advective loss from suitable nursery areas is the highest. As such, food availability is considered an important driver for larval growth and survival (Hjort, 1914; Anderson, 1988; Cushing, 1990; Houde, 2008). For cod, reproduction has evolved to ensure that spawning and larval development take place when and where conditions are favourable for larval growth, survival and ultimately recruitment as illustrated in the “match–mismatch” hypothesis (Cushing, 1990; Neuheimer *et al.,* 2018). The “match–mismatch” hypothesis explains how the overlap between larval hatching with the peak availability of plankton can significantly affect survival rates. A match scenario, where these events are synchronized, generally results in higher survival rates and stronger year classes, while a mismatch can have detrimental effects due to food limitation.

**Fig. 1:**
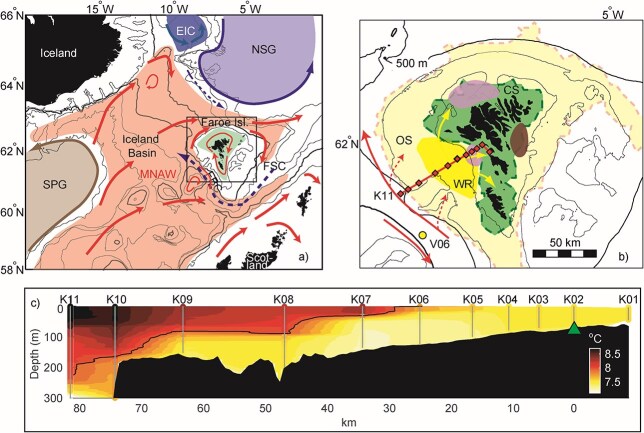
Map of the study area. (**a**) The North East Atlantic with bottom depth contours. Main warm (red arrows) and cold (blue arrows) currents carrying *C. finmarchicus* to the Faroe shelf (green) are displayed. (**b**) The Faroe shelf. The green dashed line delineates the approximate outer boundary of the CS separating the permanently mixed CS (green) from the seasonally stratified OS (light yellow) (Eliasen *et al.,* 2017b). The red solid line with black diamonds indicates the K-transect and stations with station “K11” noted. “V06” denotes the annually sampled zooplankton station in the FBC. Dark yellow indicates the western region where inflow of water and oceanic copepods is most intense, brown the pelagic 0-group cod sampling area in 2019 and blue indicates cod spawning sites. 100, 200 and 500 m bottom depth contours are indicated as solid lines. (**c**) Section of the K-transect showing temperature 20 May 2018. The 11 CTD stations are indicated with vertical lines, and the colours are interpolated temperatures, with the line indicating the 8°C isotherm. The horizontal unit is km, with 0 km at the K02 station (where the transect bends, indicated by green triangle). Abbreviations: SPG, Subpolar Gyre; MNAW, Modified North Atlantic Water; EIC, East Icelandic Current; NSG, Norwegian Sea Gyre; FBC, Faroe Bank Channel; FSC, Faroe Shetland Channel; WR, western region; CS, central shelf; OS, outer shelf.

The Faroe shelf, situated on the Iceland-Scotland Ridge (Fig. 1a), is a spawning area for several commercially important fish stocks, including cod. Recruitment to the Faroe Plateau cod stock, which is variable between years, has largely failed throughout the last two decades (ICES, 2024). The stock size of this historically highly exploited population has therefore become critically small. The spawning of Faroe Plateau cod takes place mainly from February to May (peaking in the second half of March) (Ottosen *et al.,* 2018). The main spawning grounds are in two areas, one to the west of the islands (overlapping our study area, see the Study Area section and Fig. 1b) and one to the north of the islands at bottom depths of 80–150 m (Ottosen *et al.,* 2018). These are close to the areas where the highest inflow of oceanic copepods is observed during spring (Gaard and Hansen, 2000). Despite a considerable risk of larvae drifting off the shelf, the food-related benefits of spawning in these areas may outweigh the possibility of advective loss, as seen elsewhere (e.g. Munk *et al.,* 1995). The pelagic larvae and juveniles are retained on the shelf by the shelf front and dispersed throughout the shelf with the tidal currents (Gaard and Steingrund, 2001). The spatial distribution of pelagic cod juveniles in late June, shortly before settling, is concentrated inside the 100–150 m bottom depth contour i.e. on the central shelf (CS) (Jacobsen *et al.,* 2019). Recent research has shown that the abundance of cod larvae shortly after spawning is highly correlated to the size of the spawning stock (SSB) (*r* ~ 0.9). However, this correlation decreases rapidly as the correlation between the abundance of 2–3 months old juveniles and the SSB is only 0.44, and recruitment at year 1 is not significantly related to the size of the SSB. Instead, recruitment to the Faroe Plateau cod stock is in part related to variable survival during the pelagic larval and juvenile stages (Jacobsen *et al.,* 2022a). Similar relationships have also been observed in other cod stocks (ICES, 2022).

Annual 0-group surveys conducted on the Faroe shelf in late June have revealed a positive correlation between the mean length and abundance of pelagic cod juveniles. Significant interannual variations in mean length are related to fluctuations in growth and survival during the pelagic larval and early juvenile stages. Food availability seems to be the most important factor influencing growth of Faroe Plateau cod larvae and pelagic juveniles, as there is a close relationship between primary production on the central Faroe shelf and juvenile mean length represented by a so-called 0-group length index (Jacobsen *et al.,* 2019). However, while cod juvenile mean length and abundance are positively correlated, the abundance shows considerably more variability than the mean length, indicating that factors other than the local food production may also influence survival.

Like elsewhere (Heath and Lough, 2007), the main food of pelagic Faroe Plateau cod larvae and juveniles up to 40 mm in length is zooplankton (mainly copepods). Extensive gut content studies on Faroe Plateau cod have shown that during the first-feeding period in late April, when the larvae are 3–9 mm, they feed on copepod eggs and nauplii, mainly from the oceanic copepod *Calanus finmarchicus* (Gaard and Steingrund, 2001; Jacobsen *et al.,* 2020). As the fish larvae grow, they gradually switch to larger food items. In May, when larvae/juveniles are 10–20 mm, they feed mainly on copepodites of the ubiquitous *Pseudocalanus* sp. and copepodites of both neritic (*Acartia* sp. and *Temora longicornis*) and oceanic origin (*C. finmarchicus*) (Gaard and Steingrund, 2001; Gaard and Reinert, 2002; Jacobsen *et al.,* 2020). In June, juvenile cod, with a length of 20–30 mm, feed mainly on late-stage copepodites of *C. finmarchicus*, produced during spring, while cod between 30 and 40 mm add decapod larvae to their diet. In July, at lengths of ~40 mm, the juveniles migrate towards land and start feeding on the benthos (Gaard and Reinert, 2002; Jacobsen *et al.,* 2020).

The copepod reproduction in the CS during spring and summer is generally regulated by the phytoplankton production (Gaard, 2000; Debes *et al.,* 2008a; Jacobsen *et al.,* 2022b). The phytoplankton spring bloom usually starts in May, but both the timing and intensity is highly variable between years (Eliasen *et al.,* 2017a). However, pre-bloom *C. finmarchicus* egg production may also be fuelled by internal lipid reserves from the overwintering stock (Madsen *et al.,* 2008), and a significant production of eggs is seen during the pre-bloom in April (Gaard, 2000; Debes and Eliasen, 2006). The abundance of *C. finmarchicus* in the CS requires horizontal advection, since the shelf is devoid of the species during winter (Gaard and Hansen, 2000). *Acartia* sp. together with *Pseudocalanus* sp. usually increase in abundance in May following the increase in phytoplankton biomass, although *Pseudocalanus* sp. is not found in high concentrations at any time of year (Gaard, 1999; Debes and Eliasen, 2006). The abundance of *C. finmarchicus* in the CS peaks in May and during this time it is likely affected by both the abundance in oceanic waters, the variable exchange rate between the on- and off-shelf waters and the variable production in the CS (Gaard, 2000; Gaard and Hansen, 2000; Debes *et al.,* 2008a). In June, the community is dominated by the neritic copepods *T. longicornis* and *Acartia* sp. *C. finmarchicus* abundance is usually lower in June than in May, likely due to predation by juvenile fish (Jacobsen *et al.,* 2019, 2022a).

It remains unclear why and when during the pelagic cod larva and/or juvenile development growth and survival bottleneck(s) occur. From gut content studies, it has been suggested that a bottleneck may exist in May during the beginning of the transition from the larval to the juvenile stage, when cod larvae are ~10 mm in length (Jacobsen *et al.,* 2020, 2022a). This energy demanding transition, also known as the start of metamorphosis, marks the onset of competition for food (Thompson and Harrop, 1991; Thorisson, 1994). This means that a lack of nauplii and young copepodite stages of *C. finmarchicus* and medium-sized copepods such as *Acartia* sp. and *Pseudocalanus* sp. can potentially reduce Faroe Plateau cod larval growth and survival. Here, we set out to identify when the growth and survival bottleneck(s) of pelagic 0-group cod occurs and why. Our main objective is to understand why pelagic juvenile cod abundance and mean length vary significantly between years, focusing on which prey have the most effect on the growth and survival of pelagic 0-group cod. In order to answer these questions, we analysed three contrasting years (2017–2019) of observations of pelagic 0-group cod and the seasonal abundance of their zooplankton prey groups to the west of the Faroe shelf (Fig. 1b). We look for patterns in the plankton that indicate match/mismatch (Cushing, 1990) influences on pelagic juvenile cod abundance and mean length. From there we develop the hypothesis that: *The mean length and abundance of 0-group cod are positively correlated with the spring abundance of C. finmarchicus in oceanic waters adjacent to the shelf as well as the CS plankton production*, which is tested using longer (1993–2024) cod juvenile time series from the annual 0-group survey and relevant available longer plankton series.

## MATERIALS AND METHODS

### Study area

The Faroe shelf can be divided into exclusive domains based on oceanography (Larsen *et al.,* 2008, 2009) and on phytoplankton (biomass and species composition) variability (Gaard, 1996; Eliasen *et al.,* 2017b). One major division is formed by the tidal front at the 100–150 m bottom depth band, which separates the permanently well-mixed CS from the surrounding seasonally stratified outer shelf (OS) (Fig. 1b) (Larsen *et al.,* 2009). The difference in hydrographic conditions between the CS and oceanic waters affects the composition and productivity of both phyto- and zooplankton, while the general clockwise circulation affects the distribution (Gaard, 1999; Debes *et al.,* 2008b; Jacobsen *et al.,* 2018).

The most productive region on the Faroe shelf is located in an area to the west of the islands i.e. the western region (WR, Fig. 1b). Here, the shelf is wide and tidal currents are weak, resulting in stratification of the area during spring/early summer (Eliasen *et al.,* 2017a). In the WR, spring chlorophyll-*a* (chl) means and variances are particularly high (Eliasen *et al.,* 2017a), as is *C. finmarchicus* abundance in spring (Jacobsen *et al.,* 2018), likely due to influx of oceanic and OS water to the CS through the WR (Hátún *et al.,* 2013; Rasmussen *et al.,* 2014) (Fig. 1b).

**Fig. 2:**
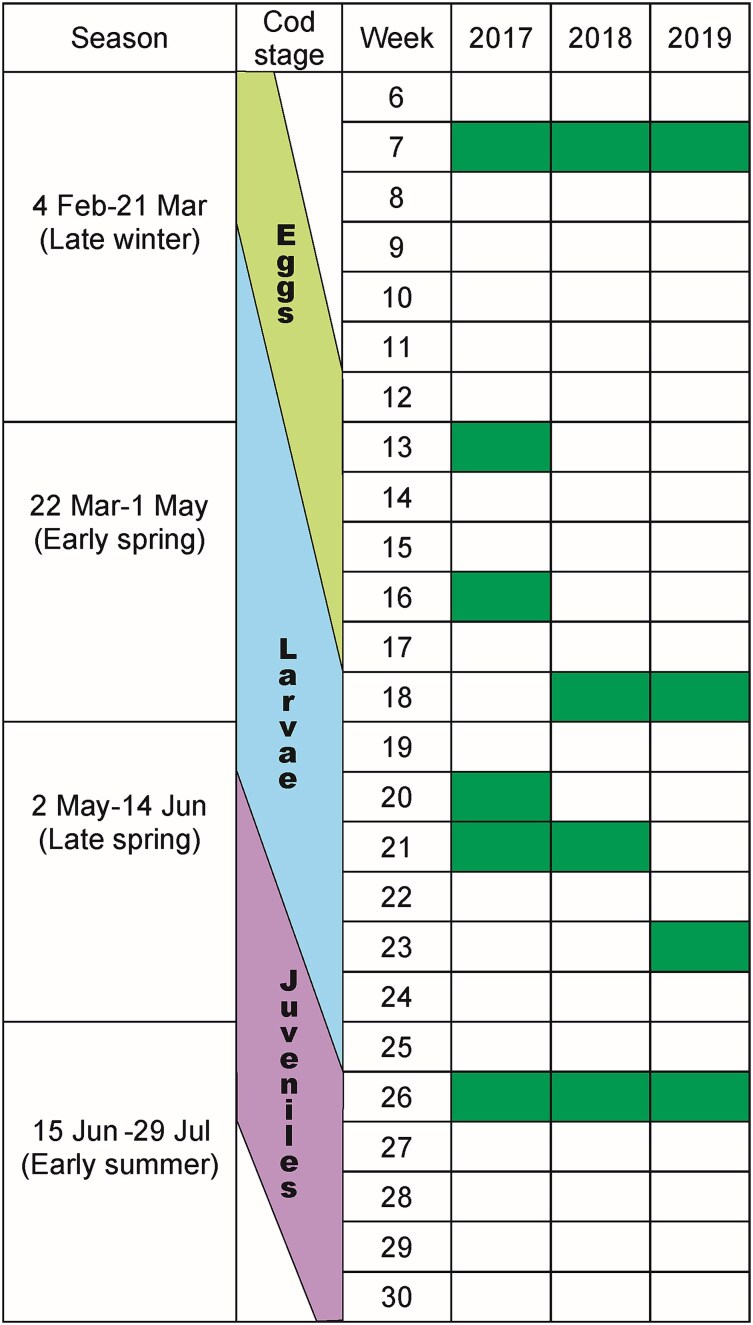
Time periods for CTD and zooplankton sampling at the K-transect 2017–2019. Dark green marks the weeks when samples were collected. Approximate timing of pelagic cod development stages are indicated.

This recognition of the WR as an important part of the Faroe shelf ecosystem led to the initiation of a new transect termed the K-transect. The main material (hydrography and zooplankton) was collected along this transect, going from the CS, through the WR and into oceanic waters, west of the Faroe Islands. The K-transect consists of 11 stations with a spacing ranging between 5 and 16 km. Station 1 is located centrally on the shelf at 60 m bottom depth and station 11 in the northern Faroe Bank Channel (FBC), where the bottom depth is 677 m. Mainly the shallow stations close to shore are short distances apart (Fig. 1b and c). Hydrographic and zooplankton data were collected four to six times from February to late June (i.e. the pelagic period of cod) during the years 2017–2019 (Fig. 2).

### Seasonal analyses

#### Cod larva and juvenile length and age in 2019

To estimate pelagic 0-group cod growth and survival throughout a season, fish larvae and juveniles were collected five times from April to June in 2019 (Table I). Sampling was done in an area on the eastern side of the CS, i.e. inside the shelf front (Fig. 1b). Still, since the entire CS has a fairly homogeneous character (Larsen *et al.,* 2008; Eliasen *et al.,* 2017b), we expect that the results represent the whole CS area.

**Table I:** *Sampling dates and data collection summary for cod larvae and juveniles collected in 2019*.

Date	Lat (^o^N)	Lon (^o^W)	Bot. depth (m)	Gear	Mesh size (mm)	Mouth opening (m)	Tow speed (ms^−1^)	Tow duration (min)
27 Aril 2019	62.17	6.34	77.5	Bongo	0.1	0.6	0.5	V-shaped haul
14 May 2019	62.03	6.42	84.5	MIK	0.5	2	1.1	15–30
14 May 2019	62.03	6.41	81.5	MIK	0.5	2	1.1	15–30
23 May 2019	61.92	6.42	96	MIK	0.5	2	1.1	15–30
23 May 2019	62.10	6.32	91	MIK	0.5	2	1.1	15–30
04 June 2019	61.92	6.42	94.5	MIK	1.5	2	1.1	15–30
04 June 2019	62.10	6.32	87.5	MIK	1.5	2	1.1	15–30
23 June 2019	61.91	6.41	94	Pelagic trawl	5	8 × 5	1.5	30

The towing depth of the MIK net and trawl was between 30 and 50 m.

Collecting pelagic 0-group cod throughout their development requires a shift in sampling gear as the population size decreases, while the individual size and swimming abilities increase. In the initial period (i.e. collecting yolk-sac and early-stage larvae) a Bongo-net with 100 μm mesh was used. The net was lowered slowly down to 50 m depth and up again, while the ship was towing at a forward speed of ~2 knots. Thus, the samples were collected as double oblique net hauls in the upper 50 m. In May and early June, a larger Method Isaac Kid (MIK) net (2 m diameter) with 0.5 mm meshes was towed at 30–40 m depth with a speed of ~2 knots. The volumes of the filtered water were measured with Hydro-Bios flow meters mounted on the net openings. In late June, a small trawl with 5 mm meshes in the cod end was towed at ~30–40 m depth with a speed of ~3 knots. A data collection summary of 0-group cod sampled in 2019 is provided in Table I.

Within a few hours of the collection, fish larvae and juveniles were sorted and preserved in 70% ethanol. In the laboratory, the fish were identified to species, enumerated and length measured to the nearest millimetres. No correction for shrinkage due to preservation was made. For age analysis purposes, sagittae and lapillae from a total of 100 larvae/juveniles were removed under a dissecting microscope with a polarized light source, and mounted on microscope slides using mounting wax. Pictures of the otoliths were taken with 20×, 40× and/or 63× magnification and calibrated with a 1 mm ruler to microns. The images were saved and otolith analyses were done using Image J. In most cases the counting was done on the lapillus due to its clearer daily rings in larvae and small juveniles, but in some cases the sagitta was used.

#### Hydrography and zooplankton along the K-transect 2017–2019

The hydrographic settings along the K-transect (Fig. 1b and c and Fig. S1) were observed using a SeaBird 911+ CTD (Conductivity, Temperature and Depth). The temperature and salinity data were calibrated, using salinity samples that were analysed with an Autosal salinometer. The CTD was also equipped with a fluorometer, but due to technical issues, some of the transects had no fluorescence measurements. The fluorescence data have not been calibrated against chl samples. All data from the CTD have been quality controlled and averaged into 1 m bin levels.

Due to these issues with the fluorometer, 8-day averaged, merged L3 chl data (CHL1) with 4.6 km spatial resolution were downloaded from http://globcolour.info (version 2018.4) (chl_sat_), and subsequently gridded onto the K-transect. Remote sensing values in the CS have been validated against *in situ* measurements of chl; although they are generally lower than the *in situ* measurements, they capture well the fluctuations observed in *in situ* observations (Eliasen *et al.,* 2017b).

Zooplankton was collected with vertical hauls from 50 m depth to the surface along the K-transect (Fig. 1b and c). A WP-2 ring net with a mesh size of 200 μm was used and the towing speed was 0.3–0.5 ms^−1^. In the laboratory, preserved zooplankton samples were purged of formaldehyde and sub-sampled with a Motoda cylinder splitter until at least 200 individuals were left for identification and enumeration.

For this work, we solely focus on zooplankton prey items that are abundant in the diet of pelagic 0-group cod, i.e. *Acartia* sp. *Pseudocalanus* sp., *T. longicornis* and *C. finmarchicus* (Jacobsen *et al.,* 2020). For *C. finmarchicus*, the abundance is differentiated into copepodite stages: CI–CII, CIII–CIV and CV–CVI. While calanoid eggs and nauplii are a large part of the cod larval diet during the first feeding stages, these are not caught effectively with the WP-2 net with 200 μm meshes, and they are therefore not included in our study.

Due to somewhat irregular temporal sampling intervals, the zooplankton data were grouped into time periods based on the equinox and solstice in order to enable seasonal comparisons of zooplankton composition between years (Fig. 2). If more than one sample was taken in a time period, mean abundances were calculated. In order to discriminate community differences of the zooplankton, multidimensional scaling was applied in conjunction with the Bray–Curtis dissimilarity index of the six prey groups of interest for each period separately. Temporal patterns were assessed through careful visual inspection, since statistical analyses were impractical given the limited number of observations.

### Interannual analyses

Interannual variability in pelagic juvenile cod abundance and mean length was based on all stations inside the 100 m depth contour sampled on the mentioned annual 0-group survey, which has been conducted in the second half of June since 1983 (Jacobsen *et al.,* 2019). This is towards the end of the pelagic phase (i.e. close to settlement) of juvenile cod (Fig. 2). For a thorough description of how juvenile length and abundances are sampled and calculated, refer to Jacobsen *et al.,* 2019. Differences in abundance and mean length during the years 2017–2019 were estimated using Kruskal–Wallis tests.

Interannual variability in oceanic abundances of the six previously mentioned prey groups was based on samples collected annually in mid-May (sometimes, early June) (i.e. late spring) 1993–2024 at station V06 located in the FBC near the offshore end of the K-transect (Fig. 1b). The bottom depth at V06 is 885 m. Sample collection and analysis was as described in the Hydrography and Zooplankton along the K-transect 2017–2019 section and a sample collection summary is provided in the supplementary material (Table S2). These data are novel and have not been published before.

The primary production in the CS is already known to have significant influence on higher trophic level species on the Faroe shelf, including 0-group fish (Eliasen *et al.,* 2011; Jacobsen *et al.,* 2019). We therefore utilize the CS primary production index (PPI), which is a proxy for new primary production in the CS from spring to mid-summer (end of June), as an indicator for food production in the CS. The series was developed in the 1990s (Gaard *et al.,* 1998) and has been used in several publications since (e.g. Gaard *et al.,* 2002; Steingrund and Gaard, 2005; Bonitz *et al.,* 2018; Matras *et al.,* 2022).

The time series data are included in the supplementary material (Table S3).

### Statistical analyses

To test our hypothesis, partial least square regression (PLS-R) was performed with juvenile cod mean length and juvenile cod abundance as response variables, respectively, and the PPI and copepod abundances of the six prey groups in oceanic waters in May as predictor variables. PLS-R was selected because several of the predictor variables exhibit collinearity. This multivariate statistical technique, which merges elements from principal components analysis and multiple regression, is particularly well-suited for ecological studies where predictor variables are collinear (Wold *et al.,* 2001). Cod abundances were log transformed prior to modelling to ensure that residuals met the assumptions of homoscedasticity, normality and linearity required for robust modelling. All variables were standardized to ensure comparability and prevent differences in magnitude from biasing the analysis. Leave-one-out (LOO) cross-validation was employed to select the optimal number of components based on the root mean square error of prediction. Model residuals were tested for normality using the Shapiro–Wilk test, and diagnostic plots were inspected to assess model assumptions. To identify the most significant predictors, the variable importance of projection (VIP) scores in the PLS-R models were used to assess the importance of each variable in the response variables. A VIP >1 was considered to have a statistically significant effect on the response variable (Chong and Jun, 2005). Data analyses were implemented using the statistical software R, version 4.3.2 (R Core Team, 2023).

## RESULTS

### Seasonal patterns of cod, zooplankton and hydrography

**Fig. 3:**
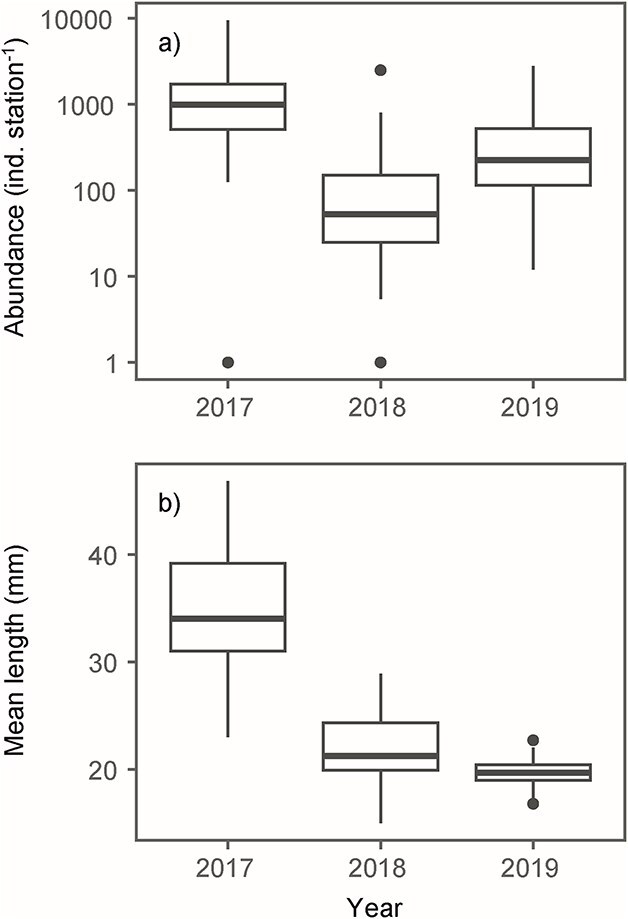
Boxplot of cod juvenile (**a**) abundance (log) and (**b**) mean length (mm) in the second half of June 2017–2019. The plot is based on the abundance and mean length of all stations inside the 100 m bottom depth contour.

The pelagic juvenile cod abundance in the second half of June was significantly different between the years 2017–2019 (χ^2^ = 56.99, 𝑑𝑓 = 2, *n* = 131, *P* < 0.001). Similarly, the mean lengths were also significantly different between years (χ^2^ = 86.58, 𝑑𝑓 = 2, *n* = 126, *P* <  0.001). The abundance was highest in 2017 (~1300 ind. station^−1^) and lowest in 2018 (~150 ind. station^−1^) (Fig. 3a). The mean length was ~50% higher in 2017 (35 mm) than in 2018 and 2019 (21.8 and 19.7 mm, respectively) (Fig. 3b).

**Fig. 4:**
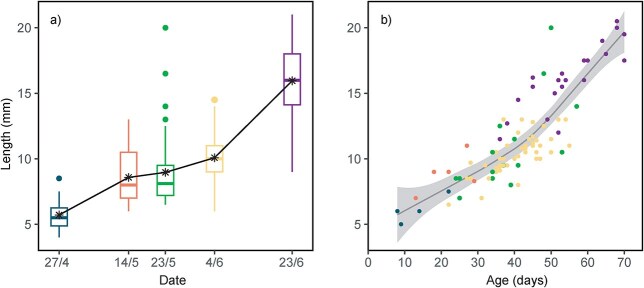
(**a**) Boxplot of length of cod sampled in 2019. Stars and solid line represent mean lengths. (**b**) Relationship between body length (mm) and age (days) in 2019. Colours indicate the time of sampling as shown in Fig. 4a. The solid line represents a LOESS fit and the grey area the associated 95% confidence interval.

The mean length of preserved pelagic 0-group cod in 2019 increased from 5.7 mm on 27 April to 16.0 mm on 23 June (Table II, Fig. 4). The increase in mean length was steeper from 27 April to 14 May and from 4 June to 23 June than during the period from 14 May to 4 June (Fig. 4a). The oldest fish sampled during the 0-group survey in 2019 on 23 June were ~70 days old (Fig. 4b). Mean back-calculated hatch dates for cod sampled in April and May were in mid-April, while mean hatch dates for cod sampled in June were dated to late April (Table II). This indicates that the oldest fish disappear from the cohort in late May coinciding with a prolonged period of poor growth.

**Table II:** Mean length (with standard deviation) and mean hatch dates in 2019.

Sampling date	Mean length (mm)	Mean hatch date
27 April 2019	5.7 ± 1.4 (12)	11 April 2019 (5)
14 May 2019	8.6 ± 2.1 (17)	19 April 2019 (6)
23 May 2019	9.0 ± 2.7 (45)	14 April 2019 (16)
04 June 2019	10.1 ± 1.8 (149)	24 April 2019 (49)
23 June 2019	16.0 ± 2.9 (46)	28 April 2019 (24)

Number of observations (*n*) is in brackets.

The hydrographic character of the oceanic water stations (K09–K11) was clearly different from the CS stations (K01–K03). The stations in between (K04–K08) represent the OS/frontal area, which changes signature throughout the year (Fig. S1). Multidimensional scaling analysis of the six zooplankton groups, illustrated by distribution on two-dimensional scale plots, also reveals a clear distinction between the oceanic waters and the CS (Fig. S2).

**Fig. 5:**
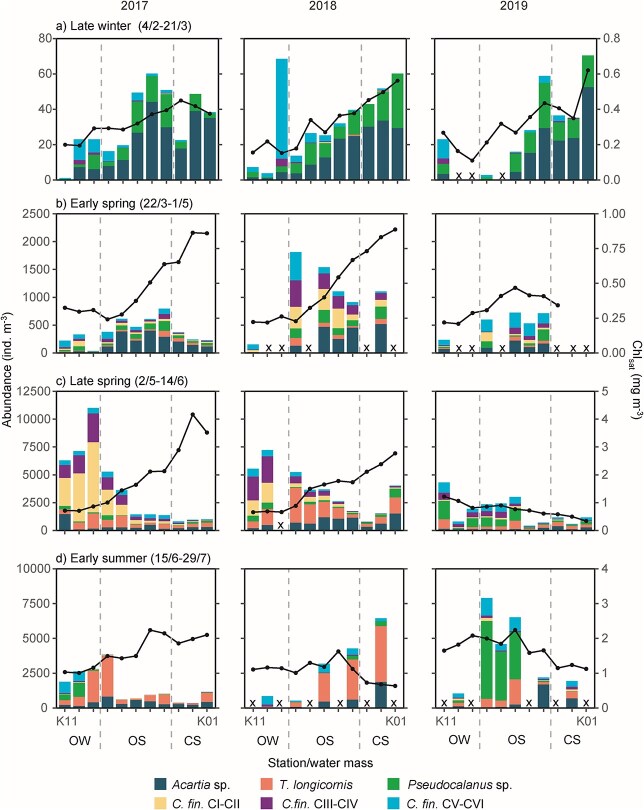
Zooplankton abundance (bars) and mean chl_sat_ (black lines) at the K-transect during (**a**) late winter, (**b**) early spring, (**c**) late spring and (**d**) early summer 2017–2019. The grey dashed lines indicate separation between oceanic waters, OS and CS, respectively. Crosses (x) indicate that no sample was taken. Abbreviations: OW, oceanic waters; OS, outer shelf; CS, central shelf. The different seasons have different y-scales to enhance readability of patterns across the section, while the different years in each season have fixed y-scales to enhance comparability between years.

In late winter (4 February to 21 March), zooplankton as well as chl_sat_ values were generally low, but there was a pattern of more plankton closer to the islands all years. Zooplankton abundances and chl_sat_ values appeared positively linked, with more zooplankton on the shallower side. *Acartia* sp. followed by *Pseudocalanus* sp. and *C. finmarchicus* CV–CVI were the most abundant copepods (Fig. 5a).

In early spring (22 March to 1 May), zooplankton and chl_sat_ values were higher than in late winter throughout the transect. Zooplankton abundances were the highest in the OS stations, but chl_sat_ values in 2017 and 2018 were higher closer to land. *Acartia* sp. and a mixture of *C. finmarchicus* stages were the most abundant copepods. In addition, chl_sat_ values were slightly higher in oceanic waters in 2017 than in 2018 and 2019 (Fig. 5b).

In late spring (2 May to 14 June), zooplankton abundances peaked in oceanic waters. Chl_sat_ values were similar in oceanic waters between years, but in the CS the values were very high in 2017 and very low in 2019. There was a clear distinction between zooplankton abundances at oceanic stations and CS stations, with much higher abundances in oceanic waters. This was especially the case in 2017, when *C. finmarchicus* CI–CII dominated the zooplankton community in oceanic waters (Fig. 5c).

While chl_sat_ values peaked in oceanic waters in early summer (15 June to 29 July), the abundance of most copepod groups had decreased throughout the transect, compared with late spring. One exception was the abundance of *T. longicornis*, which peaked in early summer. 2017, 2018 and 2019 had very different zooplankton patterns across the transect. In 2017, abundances were clearly higher in oceanic waters than in the CS. In 2018, the situation was reversed and in 2019 abundances appeared the highest in the OS, with particularly many *Pseudocalanus* sp. For chl_sat_, the pattern was opposite. Chl_sat_ values were higher in the CS than in oceanic waters in 2017, but reverse in 2018 and 2019 (Fig. 5d). Data means (with standard deviation) for the CS and oceanic waters, respectively, are given in Table S1.

Overall, the largest differences in the plankton between the years 2017–2019 were (i) a much higher concentration of chl_sat_ in the CS during late spring and early summer in 2017 compared with 2018 and 2019 (Fig. 5c and d) and (ii) much higher abundance of *C. finmarchicus* (copepodite stages I-II) in oceanic waters in late spring 2017 than in 2018 and 2019 (Fig. 5c). This points to a bottom-up influence of oceanic *C. finmarchicus* abundance and CS primary production on juvenile cod abundance, and on the basis of this exploratory analysis, we developed the hypothesis stated in the Introduction.

### Interannual effects of plankton on cod juvenile abundance and mean length

**Fig. 6:**
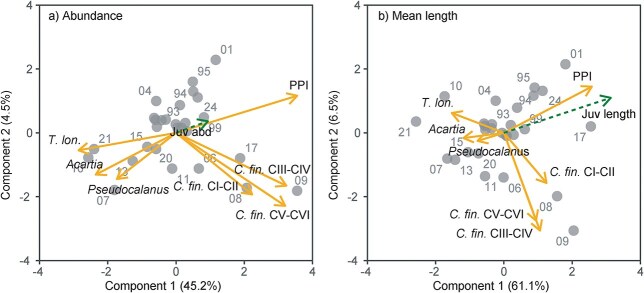
PLS-R biplots reflecting the effect of plankton variables on (**a**) cod juvenile abundance and (**b**) cod juvenile mean length 1993–2024. Solid arrows indicate explanatory variables and dashed arrow is the response. Points represent observations, i.e. years, as marked. Percentages in brackets (in the axes labels) indicate the explanatory power of the components on the response.

**Fig. 7:**
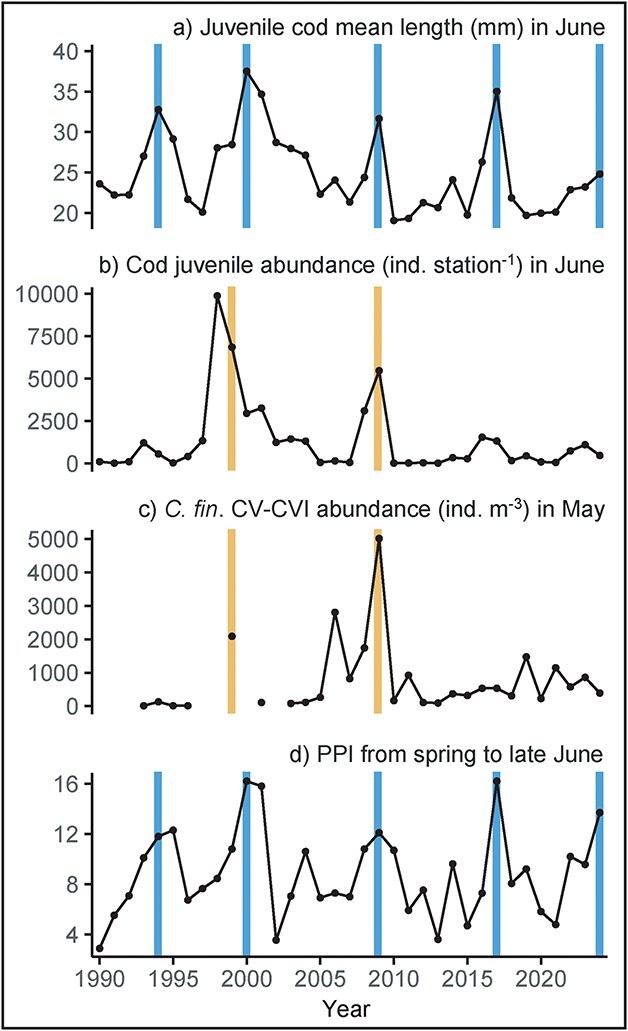
Temporal development 1990–2024 in (**a**) juvenile cod mean length (mm) in June, (**b**) juvenile cod abundance (untransformed) in June, (**c**) *C. finmarchicus* CV–CVI abundance in oceanic waters (V06) in mid-May and (**d**) the PPI from spring to late June. Vertical lines indicate simultaneous peaks in: Blue: PPI and cod juvenile mean length; Yellow: *C. finmarchicus* CV–CVI abundance and cod juvenile abundance.

For both juvenile abundance and mean length, the PLS-R models identified two optimal components through LOO cross-validation, implying that the first two components capture the most significant relationships between the predictors and the response variable (although the first component is dominant in both models) (Fig. 6a and b). VIP scores and related coefficients (Table III) from PLS-R analysis revealed that the PPI and the abundance of *C. finmarchicus* CV–CVI in oceanic waters (station V06) were key positive predictors of pelagic juvenile cod abundance. The first two principal components explained 49.7% of the variability in juvenile cod abundance and 50.5% in variability of the predictors. For juvenile cod mean length, according to the VIP scores the PPI was the sole positive explanatory contributor. The first two principal components explained 67.6% of the variability in juvenile cod mean length and 43.2% of variability in the predictors. The residuals of the final models were normally distributed (Shapiro–Wilk tests, *P* > 0.05), and residual vs. fitted plots showed no clear patterns, confirming that the assumptions of the models were met. Time series of pelagic juvenile cod mean length and abundance, *C. finmarchicus* CV–CVI abundance and the PPI are shown in Fig. 7.

**Table III:** VIP scores and regression coefficients for all predictors in the PLS-R models.

	Cod juvenile abundance (log)		Cod juvenile mean length	
	VIP score	Coefficient	VIP score	Coefficient
PPI	1.64^*^	0.84	2.13^*^	3.37
*Acartia* sp.	0.72	−0.21	0.74	−0.88
*C. finmarchicus* CI–CII	0.61	−0.01	0.66	−0.4
*C. finmarchicus* CIII–CIV	0.89	0.18	0.58	0.45
*C. finmarchicus* CV–CVI	1.06^*^	0.38	0.49	0.26
*Pseudocalanus* sp.	0.81	−0.42	0.47	−0.52
*T. longicornis*	0.93	−0.33	0.82	−0.59

VIPs >1 indicate that the predictor variable is considered significantly important for the response variable (here indicated with a star*). Regression coefficients show the direction and magnitude of their effect. Positive coefficients indicate a positive relationship with the response, and negative coefficients indicate a negative relationship.

## DISCUSSION

In this paper, we have investigated when the growth and survival bottleneck of larval and pelagic juvenile cod occurs and which prey species influence interannual variability in juvenile abundance and mean length. The outcome shows that a growth and survival bottleneck occurs in May. Juvenile mean length strongly correlates with the CS primary production, which is consistent with Jacobsen *et al.* (2019). The statistical relationship between juvenile cod abundance and the local primary production is weaker, but increases significantly when including the oceanic abundance of *C. finmarchicus*. This is discussed below.

### The growth and survival bottleneck

2019 was characterized by low mean length and abundance of pelagic cod juveniles caught during the annual 0-group survey in June. The largest juveniles in 2019 reached only ~20 mm by 70 days of age. These are small for their age compared to a high-productive year, i.e. 2001, when pelagic juveniles around 70 days old were ~30 mm (Jacobsen *et al.,* 2019). In addition, the oldest juveniles caught in June 2001 exceeded 90 days (Jacobsen *et al.,* 2019), suggesting that both larval/juvenile growth and survival of the first spawned batches was low in 2019. The abundance and mean length in 2017 is, on the other hand, comparable to that of 2001 (Jacobsen *et al.,* 2019). We therefore postulate that overall growth and survival of the first spawned batches was higher in 2017 than in 2019, which could explain the significant difference in abundance and mean length between the years. The growth and age composition analysis of 2019 suggests that a growth and survival bottleneck occurred in mid- to late May. Since larvae can survive for up to 7 days without food (Jordaan and Brown, 2003), the essential period for critical food abundance might have been slightly earlier. Although we only have data for one year, this supports earlier hypothesis of a feeding bottleneck during May (Jacobsen *et al.,* 2020), at least during a low-productive year.

### The importance of local primary production and oceanic abundance of *C. finmarchicus*

While the strong statistical relationship between primary production in the CS and juvenile fish likely stems from production of copepods in the CS, the sparsely available data have not enabled us to confirm such bottom-up influence from the zooplankton community abundances. This limitation may be due to high turnover rates of zooplankton in the CS (production vs. predation) limiting accumulation of their abundance (e.g. Munk *et al.,* 2004), and spatial structure in this system (onto-shelf flows, fronts, etc.).

The majority of cod larvae begin feeding in April, and in addition to the importance of a match between fish larvae and their prey in May, the period shortly after the exhaustion of the yolk sac in April, could be equally important for survival (Hjort, 1914). If a survival bottleneck also exists at the onset of feeding in April, this could explain why the local primary production alone cannot account for variability in juvenile abundance, since the CS primary production is usually very low in April and early May and therefore cannot reflect feeding conditions during that time (or does so only minimally) (Eliasen *et al.,* 2017a).

The lateral gradient in *C. finmarchicus* at the K-section in 2017 suggests significant predation in the OS and especially in the CS. Notably, the high abundance of *C. finmarchicus* in May 2017, had vanished by June, suggesting a large food contribution to higher trophic levels. The phytoplankton abundance was higher in the oceanic waters during early spring 2017, compared to 2018 and 2019, which potentially lead to the increased abundance of early-stage *C. finmarchicus* in late spring. Our model identified *C. finmarchicus* CV–CVI as a significant predictor of cod juvenile abundance, while the data from K-transect in 2017–2019 indicated that stages CI–CII were important. The discrepancy may be attributed to variability in sampling times and/or variability in *C. finmarchicus* phenology (Jacobsen *et al.,* 2018), amongst other factors.

*C. finmarchicus* CV–CVI sampled in the upper layers of the FBC in mid-May likely represent a mixture of overwintered individuals (*G*_0_), and the first generation (*G*_1_) produced locally during early spring. Based on the data included in this study, it is not possible to verify whether the identified relationship between cod juvenile abundance and *C. finmarchicus* CV–CVI abundance reflects a direct influence on feeding conditions in mid-May, or, if it potentially reflects production/abundance of eggs, and thus food availability 1–2 months earlier in the season, given that development from egg to CV–CVI takes ~ 45 days at 8°C (Campbell *et al.,* 2001). A former study examining Faroe Plateau cod recruitment and feeding conditions during larval development reported that 1999 was a poor year for feeding (Jacobsen *et al.,* 2022a). This inference was based on the observation of many *C. finmarchicus* CI–CII in the guts of early larvae, which Jacobsen *et al.* (2020) deduced that cod usually select against. Based on our new findings, this deduction might need revision. Juvenile abundance in 1999 and the subsequent recruitment at year 1 were high, perhaps precisely due to the high abundance of *C. finmarchicus* CI–CII.

It has previously been hypothesized that variable advection of *C. finmarchicus* on to adjacent North Atlantic shelves during spring has a significant impact on cod larval survival in several stocks (Sundby, 2000), which seems to hold true for, e.g. North Sea cod (Beaugrand *et al.,* 2003; Beaugrand and Kirby, 2010), Norwegian-Barents Sea cod (Ferreira *et al.,* 2020) and Icelandic cod (Thorisson, 1989). While the importance of *C. finmarchicus* as prey for pelagic Faroe Plateau cod was highlighted more than two decades ago (Gaard and Steingrund, 2001; Gaard and Reinert, 2002), prevailing understanding suggested that local (CS) primary production is the sole source of high on-shelf food abundance (e.g. Gaard and Steingrund, 2001). The present study demonstrates that both advection of *C. finmarchicus* onto the shelf and the CS primary production influence larval survival of a key fish species inhabiting the Faroe shelf.

The reported abundance of pelagic cod juveniles may be influenced by the Faroe Plateau cod stock remaining at historically low levels during the past two decades. However, given the shown effects of primary production and oceanic zooplankton on cod juvenile abundance, further research is needed to investigate populations in the oceanic waters surrounding the CS, and the variability in advection of oceanic waters onto the shelf and its impacts on fish recruitment. This work focused on the western shelf, which is undoubtedly crucial for phytoplankton production, inflow of oceanic waters and influence on the Faroe shelf ecosystem. However, other areas, such as the northern and eastern regions, may also be important. Furthermore, since the abundance of the most common 0-group species on the CS co-vary (Jacobsen *et al.,* 2019), plankton production in oceanic waters likely affects these species as well, including the forage fish species sandeel and Norway pout.

## CONCLUSION

A cod larva growth and survival bottleneck is here identified to be in May, on the Faroe shelf. While phytoplankton production (likely via copepod production) in the central Faroe shelf is essential for larval growth, our study suggests that in addition to this local production, oceanic abundance of *C. finmarchicus* in spring is also critical for cod larval survival. To increase the understanding of the central Faroe shelf ecosystem dynamics, a higher effort towards plankton sampling in oceanic waters surrounding the Faroes during spring is warranted, and more knowledge on temporal and spatial variability in advection of oceanic water onto the shelf and its effects on the ecosystem is called for.

## Supplementary Material

Supplementary_fbaf018

## Data Availability

Some of the data used in this study is available in the Supplementary. Other used datasets and the R script can be provided upon request to the corresponding author.
