# Supplementary material for: Identifying a growth and survival bottleneck: oceanic zooplankton abundance and Faroe shelf primary production jointly influence the survival of Faroe Plateau cod larvae
Source: J Plankton Res. 2025 May 15;47(3):fbaf018. doi: 10.1093/plankt/fbaf018 (PMC12078929; doi:10.1093/plankt/fbaf018)
Supplement: Supplementary_fbaf018 [file supplementary_fbaf018.docx]

**SUPPLEMENTARY**

**
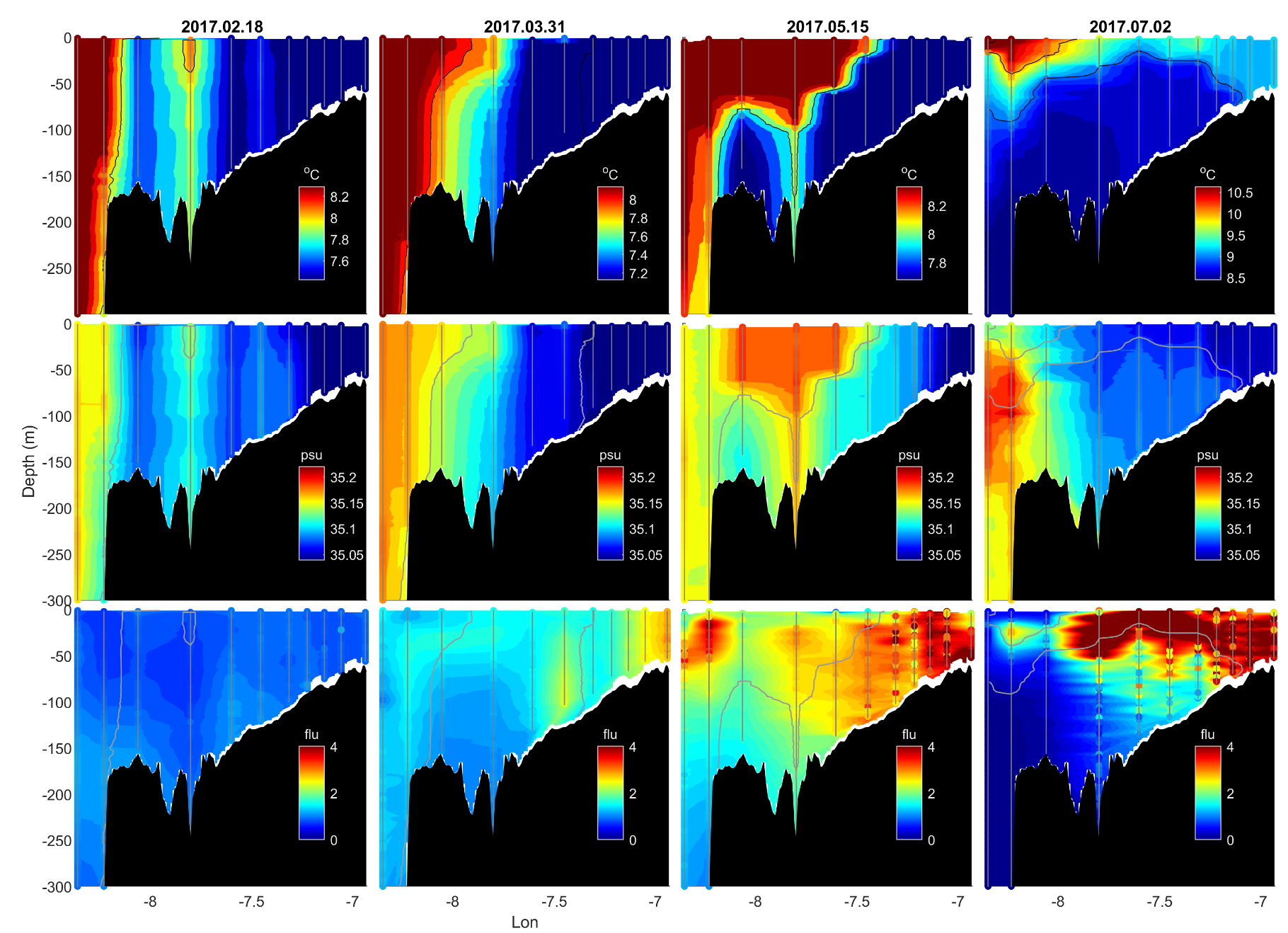
**

**
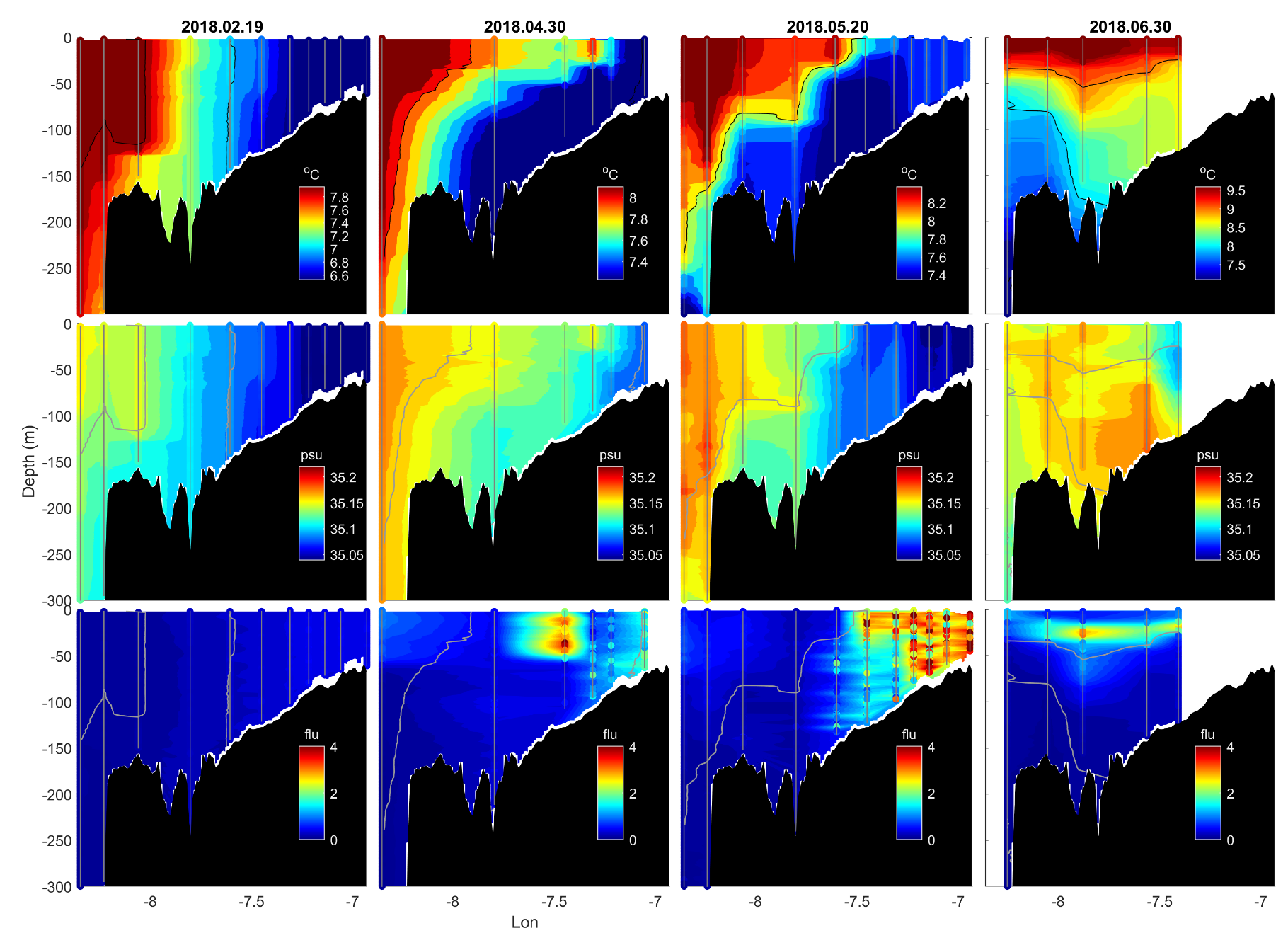
**

**
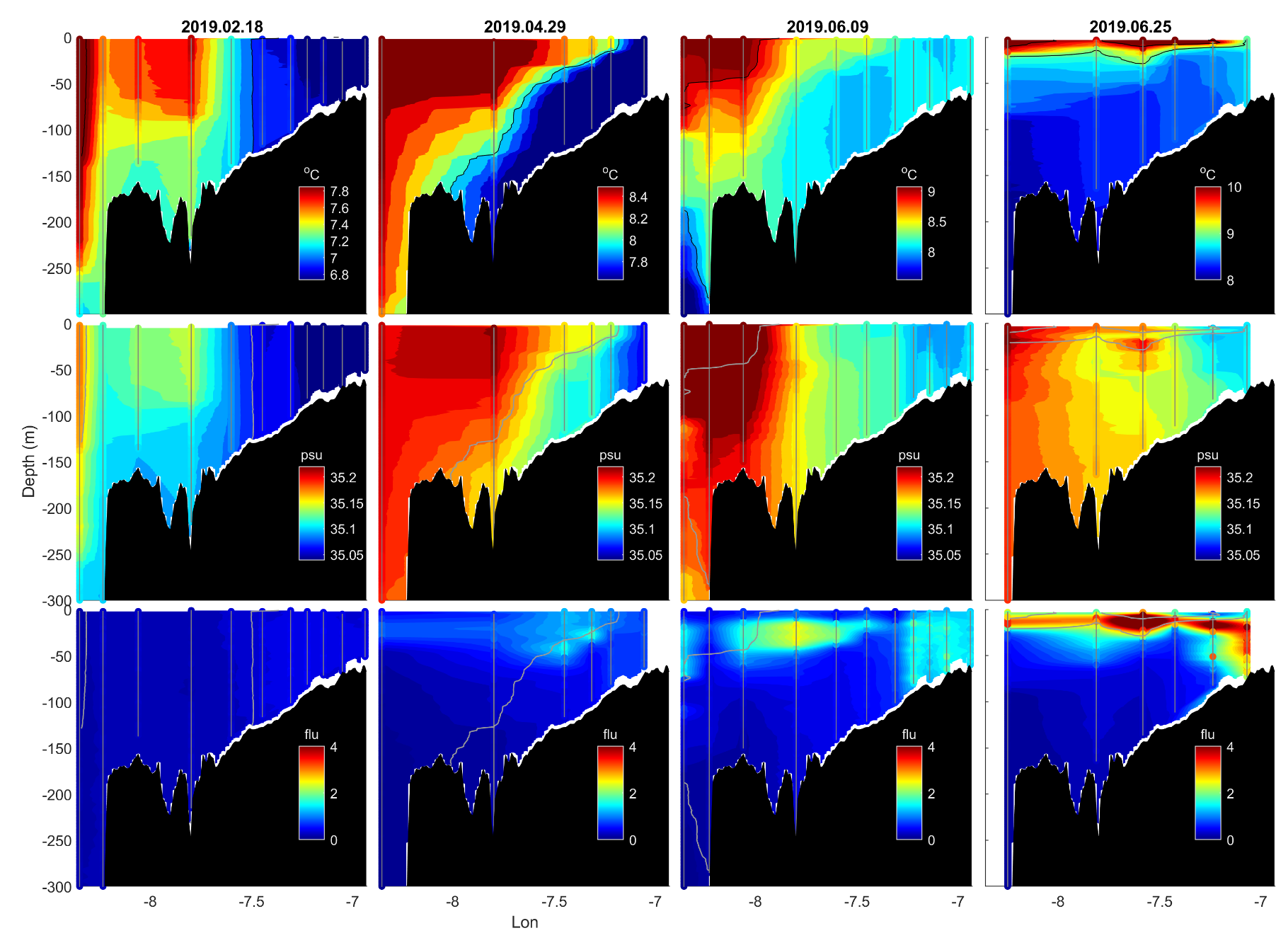
**

Fig. S1. Spatial, gridded sampling of CTD stations (vertical grey lines, see Fig. 1C for station legends) along the K-transect in late winter, early spring, late spring and early summer 2017, 2018 and 2019, respectively. Upper panels: Temperature with black isotherms plotted for each degree. Middle panels: Salinity with temperature shown with light grey lines. Bottom panels: Fluorescence with temperature shown with light grey lines.


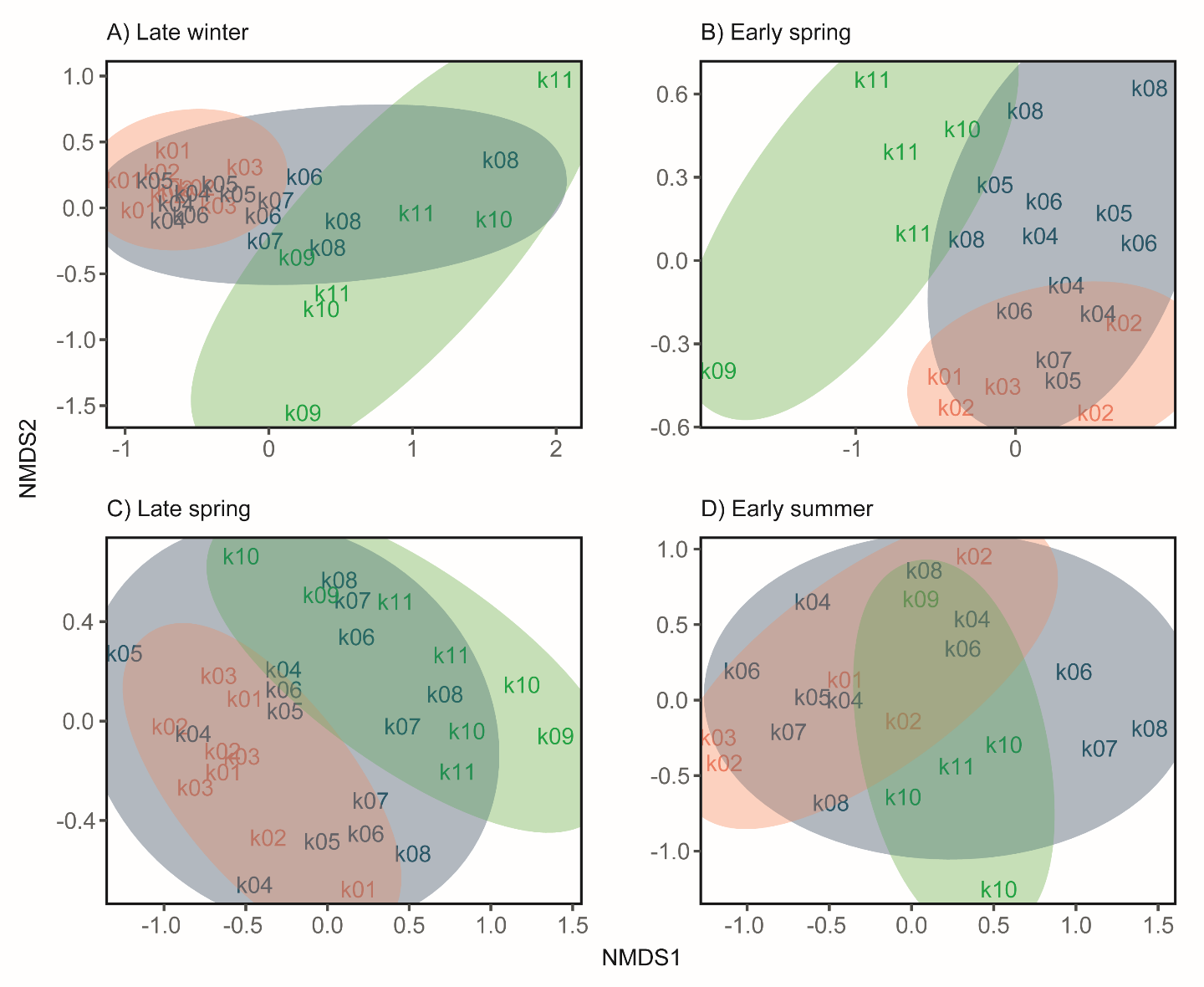


Fig. S2. Multidimensional scaling plots of six zooplankton prey groups at the K-transect during A) late winter, B) early spring, C) late spring and D) early summer 2017-2019. Red: Central shelf stations (K01-K03), blue: Outer shelf stations (K04-K08) and green: Oceanic waters stations (K09-K11).

Table S1. Mean abundance (with standard deviation) of copepods and chl_sat_ at the central shelf (K01-K03) and oceanic waters (K09-K11) in 2017-2019 (shown in Fig. 5).

|  |  | Central shelf (K01-K03) | | | Oceanic waters (K09-K11) | | |
| --- | --- | --- | --- | --- | --- | --- | --- |
| Species | Season | 2017 | 2018 | 2019 | 2017 | 2018 | 2019 |
| Chl_sat_ | Late winter | 0.4 (±0.0) | 0.5 (±0.1) | 0.4 (±0.1) | 0.2 (±0.1) | 0.2 (±0.1) | 0.2 (±0.1) |
| *Acartia* sp. | Late winter | 30.7 (±11.3) | 31 (±2.2) | 32.9 (±17) | 4.5 (±3.7) | 2.4 (±1.8) | 3.5 (±NA) |
| *C. fin.* CI-CII | Late winter | 0 | 0.3 (±NA) | 0 | 0 | 0.1 (±NA) | 0 |
| *C. fin.* CIII-CIV | Late winter | 0 | 0.3 (±NA) | 0.6 (±0) | 1.3 (±1.2) | 4.5 (±NA) | 2.9 (±NA) |
| *C. fin.* CV-CVI | Late winter | 0.8 (±0.3) | 1 (±NA) | 3.2 (±NA) | 6.5 (±5.6) | 20.5 (±31.1) | 10.9 (±NA) |
| *Pseudocalanus* sp. | Late winter | 5.3 (±3.8) | 20.1 (±9.4) | 13 (±4.3) | 3.4 (±4.3) | 2.2 (±1.5) | 5.8 (±NA) |
| *T. longicornis* | Late winter | 0 | 0 | 0 | 0 | 0 | 0 |
| Chl_sat_ | Early spring | 0.8 (±0.2) | 0.8 (±0.3) | 0.3 (±0.1) | 0.3 (±0.1) | 0.2 (±0.1) | 0.2 (±0.1) |
| *Acartia* sp. | Early spring | 156.8 (±131.5) | 517.1 (±NA) | 363.5 (±NA) | 15 (±22.5) | 12.8 (±NA) | 74.2 (±NA) |
| *C. fin.* CI-CII | Early spring | 19 (±13.7) | 128 (±NA) | 2.6 (±NA) | 39.2 (±69.8) | 37.1 (±NA) | 10.2 (±NA) |
| *C. fin.* CIII-CIV | Early spring | 14.4 (±12.9) | 117.8 (±NA) | 17.9 (±NA) | 24.9 (±33.6) | 9 (±NA) | 10.2 (±NA) |
| *C. fin.* CV-CVI | Early spring | 10 (±9.8) | 30.7 (±NA) | 23 (±NA) | 66.6 (±86.1) | 92.2 (±NA) | 99.8 (±NA) |
| *Pseudocalanus* sp. | Early spring | 42.6 (±31.3) | 215 (±NA) | 286.7 (±NA) | 40.6 (±61.8) | 2.6 (±NA) | 28.2 (±NA) |
| *T. longicornis* | Early spring | 37.9 (±32.9) | 97.3 (±NA) | 38.4 (±NA) | 8.3 (±8.8) | 0 | 12.9 (±NA) |
| Chl_sat_ | Late spring | 3.5 (±1.2) | 2.4 (±1.1) | 0.5 (±0.2) | 0.8 (±0.1) | 0.7 (±0.2) | 1.0 (±0.4) |
| *Acartia* sp. | Late spring | 280.9 (±161.9) | 820.9 (±630.1) | 250.9 (±120.4) | 573.4 (±997.5) | 348.2 (±202.7) | 140 (±21.3) |
| *C. fin.* CI-CII | Late spring | 73.8 (±67) | 42.7 (±52.3) | 35.8 (±25.6) | 4382.7 (±3438.6) | 1566.7 (±304.1) | 92.2 (±17.7) |
| *C. fin.* CIII-CIV | Late spring | 69.2 (±67) | 59.7 (±55.2) | 44.4 (±29.56) | 1768.1 (±1084.5) | 2252.8 (±144.8) | 436.9 (±174.2) |
| *C. fin.* CV-CVI | Late spring | 87.9 (±83.4) | 23.9 (±24.2) | 153.6 (±124.2) | 443.7 (±230.5) | 645.1 (±101.4) | 501.8 (±383) |
| *Pseudocalanus* sp. | Late spring | 108.7 (±51.) | 394.2 (±355.2) | 174.1 (±97.7) | 174.1 (±142.3) | 563.2 (±43.4) | 843.1 (±789.9) |
| *T. longicornis* | Late spring | 303.3 (±171) | 769.7 (±591.9) | 315.7 (±54.8) | 839.7 (±1014.5) | 1013.8 (±593.7) | 344.8 (±437.4) |
| Chl_sat_ | Early summer | 2.0 (±0.7) | 0.7 (±0.1) | 1.2 (±0.4) | 1.1 (±0.5) | 1.1 (±0.2) | 1.9 (±0.7) |
| *Acartia* sp. | Early summer | 315.7 (±105.4) | 1863.7 (±NA) | 686.1 (±NA) | 256 (±135.4) | 0 | 112.6 (±NA) |
| *C. fin.* CI-CII | Early summer | 5.6 (±3.2) | 0 | 143.4 (±NA) | 20.5 (±10.2) | 20.5 (±NA) | 133.1 (±NA) |
| *C. fin.* CIII-CIV | Early summer | 7.3 (±3.2) | 0 | 184.3 (±NA) | 61.4 (±44.6) | 143.4 (±NA) | 71.7 (±NA) |
| *C. fin.* CV-CVI | Early summer | 1.9 (±0.9) | 204.8 (±NA) | 409.6 (±NA) | 747.5 (±57.9) | 624.6 (±NA) | 276.5 (±NA) |
| *Pseudocalanus* sp. | Early summer | 12.8 (±14.5) | 368.6 (±NA) | 20.5 (±NA) | 518.8 (±473.7) | 10.2 (±NA) | 174.1 (±NA) |
| *T. longicornis* | Early summer | 278.2 (±344.8) | 4014.1 (±NA) | 491.5 (±NA) | 1082 (±1023.3) | 61.4 (±NA) | 266.2 (±NA) |

Table S2. Sample collection summary of zooplankton collected in the upper layer (0-50 m) in the Faroe Bank Channel 1993-2024.

| Station name | Date | Bottom depth (m) | Latitude (^o^N) | Longitude (^o^W) |
| --- | --- | --- | --- | --- |
| V06 | 17-05-1993 | 876 | 61.27 | 8.01 |
| V05 | 17-05-1994 | 803 | 61.33 | 7.88 |
| V05 | 19-05-1995 | 806 | 61.33 | 7.88 |
| V06 | 05-05-1996 | 882 | 61.27 | 8.00 |
| V06 | 13-06-1999 | 885 | 61.27 | 8.00 |
| V06 | 06-05-2001 | 880 | 61.27 | 8.00 |
| V06 | 03-05-2003 | 875 | 61.27 | 8.02 |
| V06 | 13-06-2004 | 885 | 61.27 | 7.99 |
| V06 | 25-05-2005 | 886 | 61.27 | 7.99 |
| V06 | 12-06-2006 | 881 | 61.27 | 7.99 |
| V06 | 19-05-2007 | 881 | 61.27 | 8.00 |
| V06 | 17-05-2008 | 888 | 61.27 | 8.00 |
| V06 | 16-05-2009 | 883 | 61.27 | 8.02 |
| V06 | 15-05-2010 | 885 | 61.27 | 8.00 |
| V06 | 23-05-2011 | 890 | 61.26 | 8.00 |
| V06 | 22-05-2012 | 883 | 61.27 | 8.01 |
| V06 | 16-05-2013 | 860 | 61.26 | 8.01 |
| V06 | 16-05-2014 | 878 | 61.27 | 7.98 |
| V06 | 24-05-2015 | 886 | 61.26 | 8.00 |
| V06 | 10-06-2016 | 884 | 61.27 | 8.01 |
| V06 | 18-05-2017 | 883 | 61.27 | 8.00 |
| V06 | 21-05-2018 | 884 | 61.26 | 7.98 |
| V06 | 10-06-2019 | 888 | 61.26 | 7.99 |
| V06 | 22-05-2020 | 879 | 61.27 | 8.02 |
| V06 | 13-06-2021 | 874 | 61.27 | 7.97 |
| V05 | 02-06-2022 | 804 | 61.34 | 7.89 |
| V06 | 20-05-2023 | 868 | 61.27 | 7.99 |
| V06 | 18-05-2024 | 880 | 61.26 | 7.99 |

Table S3. Time series used in the study (Fig. 6 and 7). Pelagic juvenile cod abundance and mean length based on mean from stations within the 100 m bottom depth contour. The PPI refers to the primary production index on the central shelf. The copepod groups show abundances in oceanic waters i.e. station V06 in May.

| Year | Cod abd. | Cod length | PPI | *Acartia* sp. | *C. fin*. CI-CII | *C. fin*. CIII-CIV | *C. fin*. CV-CVI | *Pseudocalalnus* sp. | *T. longicornis* |
| --- | --- | --- | --- | --- | --- | --- | --- | --- | --- |
| 1990 | 105.2 | 23.6 | 2.9 | - | - | - | - | - | - |
| 1991 | 17.6 | 22.2 | 5.5 | - | - | - | - | - | - |
| 1992 | 103.8 | 22.2 | 7.1 | - | - | - | - | - | - |
| 1993 | 1212.8 | 27.0 | 10.1 | 0.0 | 378.9 | 169.0 | 10.2 | 51.2 | 0.0 |
| 1994 | 557.3 | 32.8 | 11.8 | 0.0 | 394.2 | 76.8 | 128.0 | 15.4 | 0.0 |
| 1995 | 34.1 | 29.1 | 12.3 | 9.0 | 42.9 | 10.9 | 9.0 | 0.6 | 0.6 |
| 1996 | 414.8 | 21.7 | 6.7 | 5.8 | 32.0 | 13.8 | 11.5 | 0.0 | 0.0 |
| 1997 | 1340.8 | 20.1 | 7.7 | - | - | - | - | - | - |
| 1998 | 9871.4 | 28.0 | 8.5 | - | - | - | - | - | - |
| 1999 | 6848.4 | 28.4 | 10.8 | 348.1 | 491.6 | 952.3 | 2089.0 | 286.7 | 71.7 |
| 2000 | 2950.9 | 37.5 | 16.2 | - | - | - | - | - | - |
| 2001 | 3260.7 | 34.7 | 15.8 | 17.9 | 76.8 | 2.6 | 107.5 | 61.4 | 0.0 |
| 2002 | 1238.9 | 28.7 | 3.6 | - | - | - | - | - | - |
| 2003 | 1442.4 | 28.0 | 7.1 | 145.9 | 30.7 | 15.4 | 79.4 | 43.5 | 12.8 |
| 2004 | 1314.2 | 27.1 | 10.6 | 829.4 | 102.4 | 276.5 | 112.6 | 348.2 | 133.1 |
| 2005 | 57.5 | 22.3 | 6.9 | 56.3 | 133.1 | 217.6 | 261.1 | 140.8 | 7.7 |
| 2006 | 149.4 | 24.0 | 7.3 | 102.4 | 798.7 | 2007.1 | 2805.7 | 512.0 | 61.5 |
| 2007 | 53.4 | 21.3 | 7.0 | 1515.5 | 1433.6 | 1638.4 | 819.2 | 1433.6 | 0.0 |
| 2008 | 3102.8 | 24.4 | 10.8 | 266.3 | 3522.6 | 3768.3 | 1740.8 | 102.4 | 122.9 |
| 2009 | 5462.1 | 31.6 | 12.1 | 512.0 | 2273.3 | 5611.5 | 5017.6 | 307.2 | 0.0 |
| 2010 | 19.8 | 19.1 | 10.7 | 1966.1 | 2293.8 | 1024.0 | 163.8 | 1310.7 | 3235.8 |
| 2011 | 27.1 | 19.3 | 5.9 | 286.7 | 1126.4 | 1986.6 | 921.6 | 184.3 | 102.4 |
| 2012 | 41.0 | 21.3 | 7.5 | 25.6 | 138.2 | 184.3 | 107.5 | 71.7 | 0.0 |
| 2013 | 17.7 | 20.6 | 3.6 | 266.2 | 809.0 | 460.8 | 92.2 | 174.1 | 20.5 |
| 2014 | 339.3 | 24.1 | 9.6 | 184.3 | 798.7 | 778.2 | 368.6 | 286.7 | 41.0 |
| 2015 | 280.1 | 19.7 | 4.7 | 102.4 | 368.6 | 553.0 | 317.4 | 71.7 | 71.7 |
| 2016 | 1546.4 | 26.3 | 7.3 | 41.0 | 61.4 | 204.8 | 532.5 | 20.5 | 61.4 |
| 2017 | 1320.7 | 35.0 | 16.2 | 307.2 | 4608.0 | 2027.5 | 532.5 | 573.4 | 409.6 |
| 2018 | 166.8 | 21.8 | 8.1 | 20.5 | 368.6 | 737.3 | 307.2 | 0.0 | 20.5 |
| 2019 | 444.6 | 19.7 | 9.2 | 348.2 | 143.4 | 1065.0 | 1474.6 | 245.8 | 81.9 |
| 2020 | 90.6 | 20.0 | 5.8 | 215.0 | 706.6 | 962.6 | 225.3 | 112.6 | 0.0 |
| 2021 | 56.6 | 20.1 | 4.8 | 532.5 | 245.8 | 1228.8 | 1146.9 | 204.8 | 5120.0 |
| 2022 | 740.9 | 22.9 | 10.2 | 348.2 | 798.7 | 1761.3 | 573.4 | 204.8 | 450.6 |
| 2023 | 1100.6 | 23.2 | 9.6 | 593.9 | 819.2 | 1228.8 | 860.2 | 204.8 | 41.0 |
| 2024 | 469.1 | 24.8 | 13.7 | 204.8 | 634.9 | 532.5 | 389.1 | 389.1 | 122.9 |
